# Reduced Protein Synthesis Fidelity Inhibits Flagellar Biosynthesis and Motility

**DOI:** 10.1038/srep30960

**Published:** 2016-07-29

**Authors:** Yongqiang Fan, Christopher R. Evans, Jiqiang Ling

**Affiliations:** 1 Department of Microbiology and Molecular Genetics, McGovern Medical School, University of Texas Health Science Center, Houston, TX 77030, USA; 2 Graduate School of Biomedical Sciences, Houston, TX 77030, USA

## Abstract

Accurate translation of the genetic information from DNA to protein is maintained by multiple quality control steps from bacteria to mammals. Genetic and environmental alterations have been shown to compromise translational quality control and reduce fidelity during protein synthesis. The physiological impact of increased translational errors is not fully understood. While generally considered harmful, translational errors have recently been shown to benefit cells under certain stress conditions. In this work, we describe a novel regulatory pathway in which reduced translational fidelity downregulates expression of flagellar genes and suppresses bacterial motility. Electron microscopy imaging shows that the error-prone *Escherichia coli* strain lacks mature flagella. Further genetic analyses reveal that translational errors upregulate expression of a small RNA DsrA through enhancing its transcription, and deleting DsrA from the error-prone strain restores motility. DsrA regulates expression of H-NS and RpoS, both of which regulate flagellar genes. We demonstrate that an increased level of DsrA in the error-prone strain suppresses motility through the H-NS pathway. Our work suggests that bacteria are capable of switching on and off the flagellar system by altering translational fidelity, which may serve as a previously unknown mechanism to improve fitness in response to environmental cues.

The genetic information is passed from DNA to RNA to protein with high fidelity. On average, amino acid misincorporation rate is approximately 10^−3^–10^−4^ 1,2. Such fidelity is maintained at every step during gene expression via careful selection of cognate substrates and proofreading of incorrect products^3^,^4^,^5^. For example, translation of mRNA into protein requires accurate ligation of amino acids to the right transfer RNAs (tRNAs) by aminoacyl-tRNA synthetases^6^,^7^, delivery of proper aminoacyl-tRNAs to the ribosome by elongation factors^8^, and precise matching of codon and anticodon on the ribosome^9^. Despite such extensive quality control mechanisms, increased translational errors (mistranslation) are known to be caused by genetic mutations^10^,^11^,^12^, nutrient starvation^13^,^14^, aminoglycoside antibiotics^15^,^16^, oxidative stress^17^,^18^,^19^, ethanol stress^20^, and temperature shift^21^,^22^. Severe mistranslation causes global protein misfolding and aggregation^23^,^24^, which leads to cell death, mitochondrial defects, and neurodegeneration^25^. A recent study also suggests that maintaining translational fidelity is critical for bacterial stringent response^26^. On the other hand, some levels of mistranslation are tolerated and even beneficial under defined stress conditions^27^,^28^. For example, we have recently shown that increased translational errors in *Escherichia coli* improve survival under oxidative stress conditions through activation of the general stress response, which is controlled by sigma factor RpoS^29^.

Flagella are complex molecular machines critical for cell motility and chemotaxis in bacteria^30^,^31^. A flagellum is composed of over 20 different structural proteins assembled to form the motor, the hook and the flagellar filament^32^,^33^. Expression of flagellar genes is highly regulated and hierarchical^34^,^35^. The master operon *flhDC* is regulated by multiple environmental cues, and in turn controls transcription of flagellar structural genes. Compared to transcriptional regulation, translational regulation of flagellar synthesis is less understood. Recent work shows that *Bacillus subtilis* requires modification of elongation factor P to efficiently translate certain flagellar proteins^36^. How flagellar synthesis is affected by translational fidelity is completely unknown. In the present work, we demonstrate that mistranslation inhibits flagellar synthesis and motility in *E. coli*. Such inhibition is independent of RpoS, but instead requires inactivation of a histone-like nucleoid structural protein H-NS, leading to reduced expression of *flhDC*. We further show that a small RNA DsrA plays a critical role in mistranslation-mediated suppression of bacterial motility.

## Results

### Mistranslation suppresses motility and flagellar assembly

**Figure 1:**
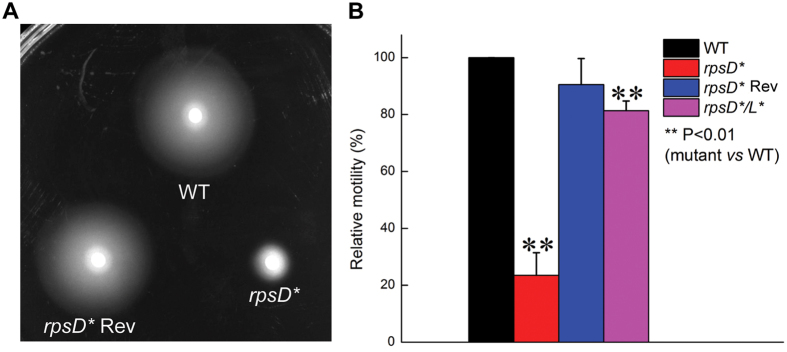
Motility defect of error-prone rpsD* strain. Motility of WT, rpsD*, rpsD* revertant, and rpsD*/L* strains were tested on soft-agar plates. In panel B, relative motility was calculated as the percentage of the spot diameter relative to the WT strain. The quantitative results are the average of at least three repeats with error bars indicating standard deviations.

**Figure 2:**
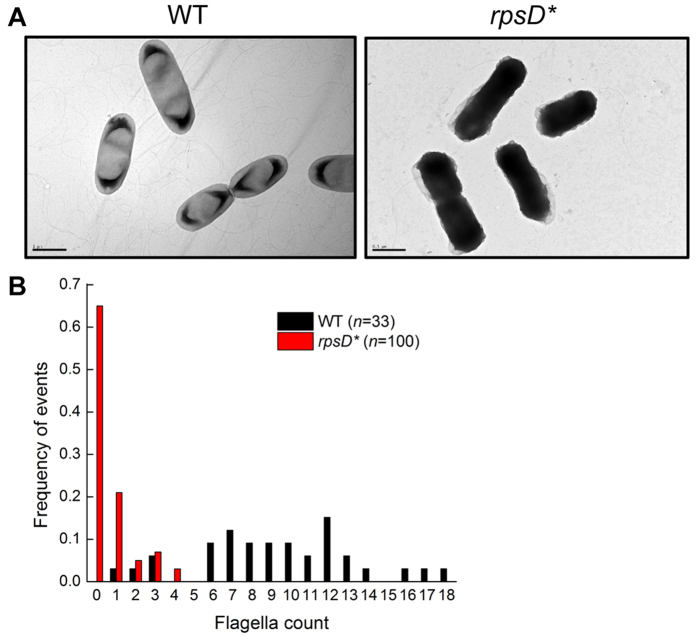
Visualization of bacterial flagellar with negative staining electron microscopy. (**A**) Representative electron microscopy views of WT and rpsD* cells. (**B**) Quantitatation and distribution of the number of flagella per cell. The rpsD* strain displayed much fewer flagella per cell compared to the WT.

To investigate the physiological impact of mistranslation, we previously engineered an *E. coli* error-prone strain by introducing a point mutation (I199N) into the chromosomal *rpsD* gene, which encodes a protein component of the ribosomal small subunit^29^. The resulting rpsD* strain (Table S1) displays 5-fold increased readthrough of the UAG stop codon compared to the parent strain MG1655, but does not show a decreased protein synthesis rate^29^. Mutations in the *rpsD* gene decrease accuracy during codon-anticodon pairing to cause global mistranslation of all mRNAs, and may decrease fidelity of initiation, elongation, and termination during protein synthesis^10^. RNA sequencing of rpsD* cells grown at 37 °C^29^ reveals that flagellar assembly is the most significantly downregulated pathway compared to wild-type (WT) MG1655 (P = 1.9 × 10^−25^). Because even WT MG1655 shows low expression of flagellar genes and slow motility at 37 °C, we tested the motility of WT and rpsD* strains at room temperature (25 °C). Our results showed that the rpsD* strain was defective in motility on soft agar plates (Fig. 1). The motility defect was rescued by either reverting the chromosomal rpsD* mutation or introducing a second mutation (K42N) in the *rpsL* gene to reduce translational errors (Fig. 1). The K42N mutation is located near the ribosomal A site and restricts pairing between codon and anticodon, and has been shown to increase decoding fidelity^37^. In addition to mistranslation caused by the rpsD* mutation, codon-specific mistranslation caused by addition of canavanine (an arginine analogue recognized by arginyl-tRNA synthetase and mistranslates arginine codons) also decreases motility (Fig. S1). Next, we used negative-staining electron microscopy to visualize the flagella of WT and rpsD* strains. Whereas WT cells contained multiple flagella per cell, most rpsD* displayed no mature flagella at all (Fig. 2). These results suggest that the motility defect caused by mistranslation is due to impaired flagellar assembly.

### Mistranslation decreases expression of flagellar genes

**Figure 3:**
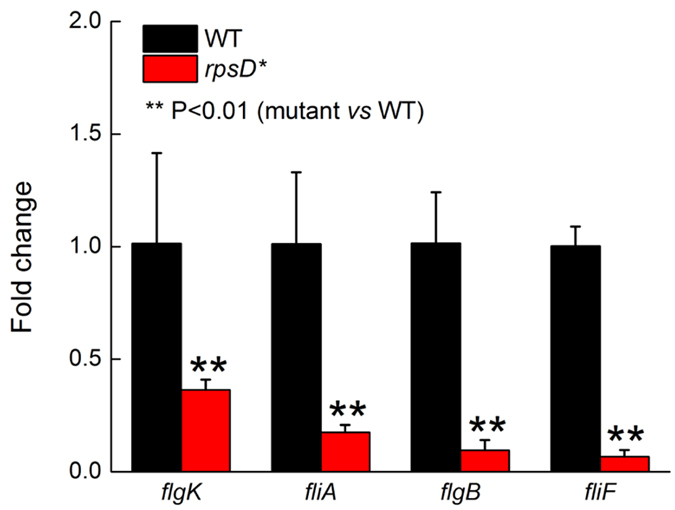
Quantitative RT-PCR of flagellar genes. All tested flagellar genes were expressed at significantly lower levels in rpsD* compared to the WT at 25 °C. The quantitative results are the average of at least three repeats with error bars indicating standard deviations.

**Figure 4:**
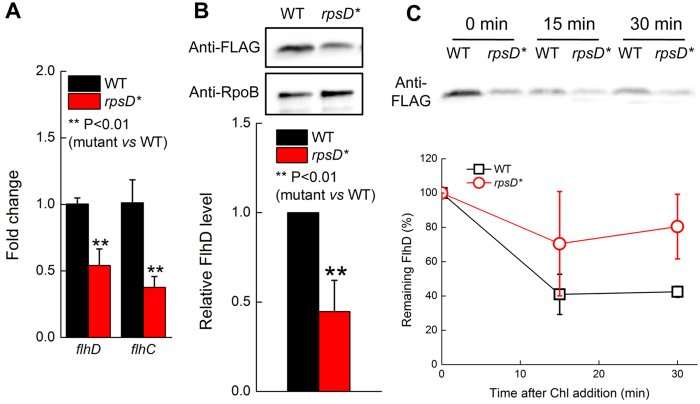
Mistranslation downregulates *flhDC* expression. (**A**) qRT-PCR of *flhD* and *flhC* mRNA. (**B**) Western blot of FLAG-FlhD protein. Quantitation of FlhD protein level is normalized with loading control RpoB. (**C**) Time course of FLAG-FlhD degradation. The quantitative results are the average of at least three repeats with error bars indicating standard deviations.

We next tested the expression levels of flagellar genes in the WT and rpsD* strains at 25 °C using quantitative reverse transcription polymerase chain reaction (qRT-PCR). The rpsD* mutation significantly decreased the mRNA levels of all tested flagellar genes, including *flgB* (encoding a flagellar basal-body rod protein), *flgK* (encoding a hook-filament junction protein), *fliA* (encoding Sigma 28 involved in synthesis of later-stage flagellar genes), *fliF* (encoding an MS-ring structural protein), and *flhDC* (encoding the master regulator of flagellar genes FlhD and FlhC) (Figs 3 and 4). Among these genes, transcription of *flgB* and *fliA* is dependent on the FlhDC complex, and *flgK* and *fliF* are controlled by both FlhDC and FliA^34^,^35^.

To determine how translational errors affect the protein level of FlhD, we inserted a Flag tag at the 3′-end of the chromosomal *flhD* gene at the native locus. Western blot using an anti-Flag antibody revealed that the FlhD protein level decreased 60% in the rpsD* strain compared to the WT (Fig. 4B). We further showed that such decrease was not due to accelerated degradation (Fig. 4C), suggesting that mistranslation downregulates FlhD at the transcriptional and/or translational level.

### Small RNA DsrA inhibits motility in error-prone strain

**Figure 5:**
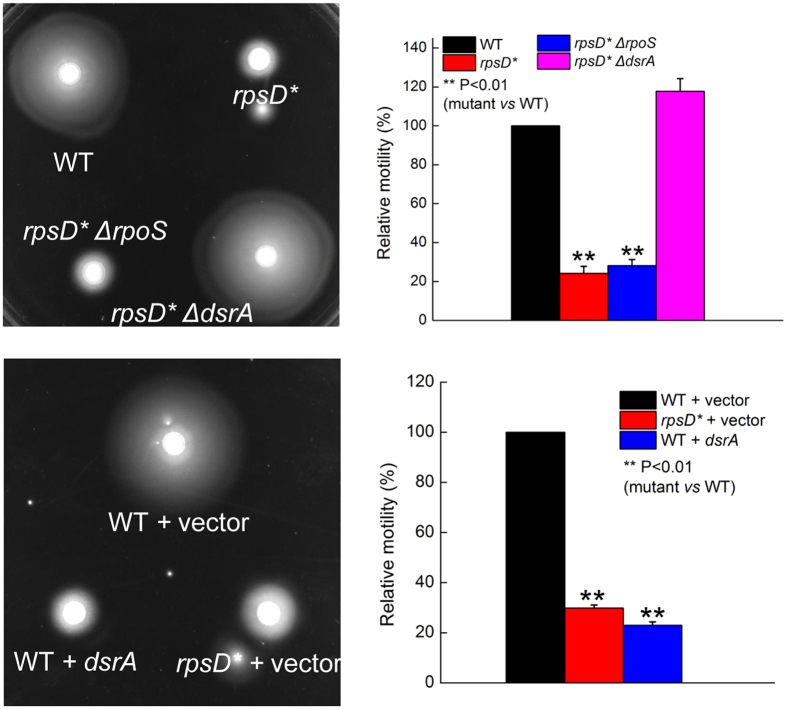
Motility of DsrA deletion and overexpression strains. Deleting DsrA restored motility in the rpsD* strain, while overexpressing DsrA suppresses motility in the WT strain. The quantitative results are the average of at least three repeats with error bars indicating standard deviations.

**Figure 6:**
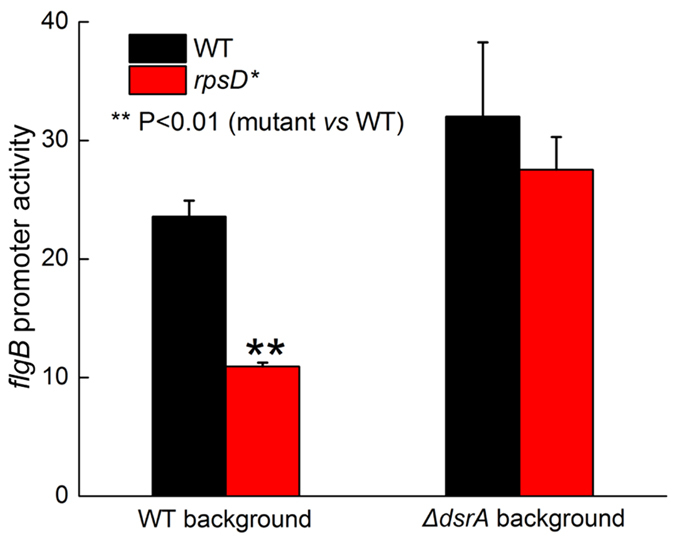
Promoter activity of *flgB*. The promoter of *E. coli flgB* was fused with *lacZ* gene on a low copy number plasmid and transformed into various *E. coli* strains. The β-galactosidase activity was determined and shown as Miller Units. The rpsD* mutation decreased *flgB* promoter activity, and deleting DsrA fully restored transcription of *flgB* promoter. The results are the average of at least three repeats with error bars indicating standard deviations.

We have previously shown that translational errors activate the general stress response, which is controlled by RpoS^29^. The increase of RpoS level under error-prone conditions at 37 °C depends on a small RNA DsrA^29^. It has been suggested that RpoS negatively regulates expression of FliA and cell motility in *E. coli*^38^. We thus tested whether mistranslation suppresses motility through upregulation of RpoS. Deleting *rpoS* in the rpsD* strain was not able to restore motility (Fig. 5), suggesting that RpoS does not play a major role in flagellar synthesis under error-prone conditions. However, deleting *dsrA* fully rescued the motility defect of the rpsD* strain (Fig. 5). Consistently, overexpressing DsrA from a plasmid in the WT strain suppressed motility (Fig. 5). To test the role of DsrA in regulating expression of flagellar genes, we constructed a *lacZ* reporter under the control of *flgB* promoter. In line with the qRT-PCR results (Fig. 3), the activity of *flgB* promoter (controlled by FlhDC) decreased 60% in the rpsD* strain compared to the WT (Fig. 6). Deleting DsrA enhanced transcription of *flgB* to almost the same level as the WT. Addition of canavanine also decreased the activity of *flgB* promoter (Fig. S1B).

**Figure 7:**
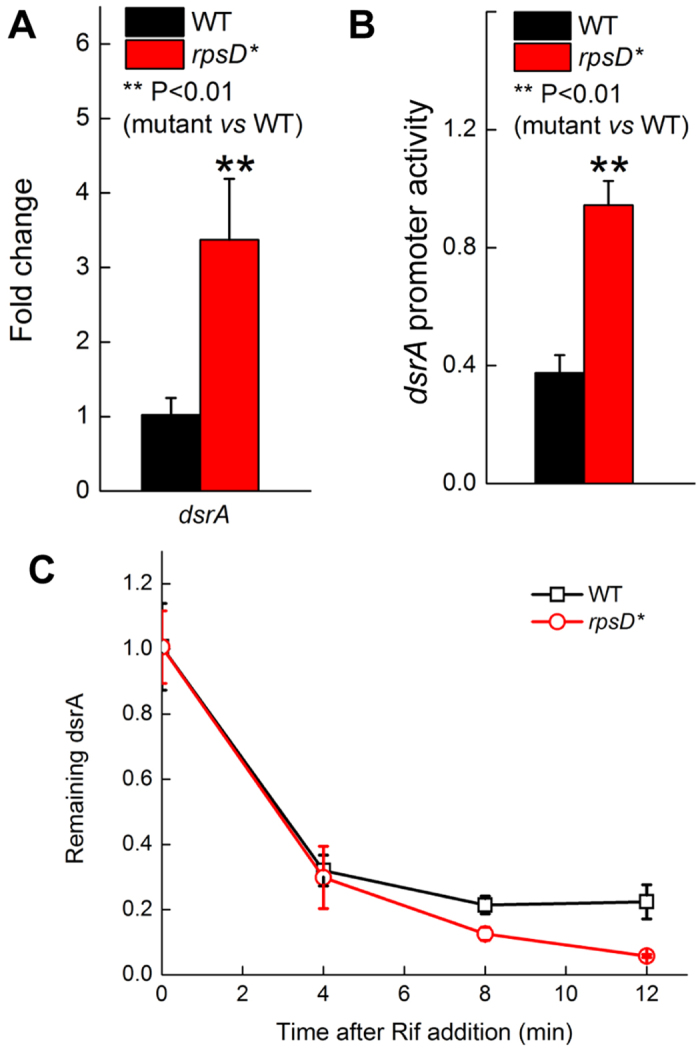
Mistranslation increases DsrA expression. (**A**) Steady state level of DsrA RNA determined by qRT-PCR. (**B**) Promoter activity of DsrA determined using a YFP reporter. (**C**) Degradation of DsrA in *E. coli* strains. The results are the average of at least three repeats with error bars indicating standard deviations.

DsrA is induced at low temperatures (e.g., at 25 °C) through enhanced transcription and improved stabilization^39^. Using qRT-PCR, we found that the RNA level of DsrA was increased 3-fold by the rpsD* mutation at 25 °C (Fig. 7A). To further investigate how mistranslation enhances DsrA level, we tested transcription of *dsrA* using a yellow fluorescent protein reporter under the control of *dsrA* promoter. Transcription of *dsrA* promoter increased 2.5-fold in the rpsD* strain compared to the WT (Fig. 7B). Next, we determined the stability of DsrA by inhibiting transcription with rifampicin (Rif) and following the RNA level over time. The rpsD* mutation did not enhance the stability of DsrA (Fig. 7C), suggesting that the increase in DsrA RNA occurred at the transcriptional level. Collectively, our data suggest that mistranslation elevates the DsrA RNA level, which in turn downregulates expression of flagellar genes and suppresses motility.

### DsrA-mediated motility suppression depends on H-NS

**Figure 8:**
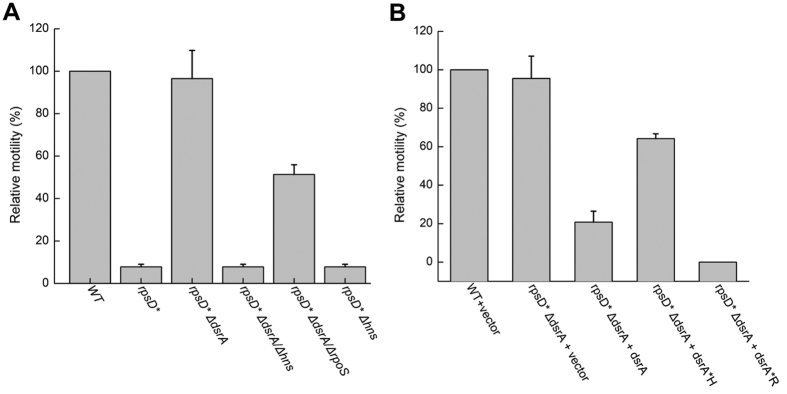
DsrA suppresses bacterial motility through H-NS. (**A**) Deleting H-NS abolishes the effect of DsrA deletion that restores motility in rpsD* cells. (**B**) Overexpressing H-NS specific DsrA (*dsrA* *R) suppressed motility in WT cells. The results are the average of at least three repeats with error bars indicating standard deviations.

In addition to RpoS, another major target regulated by DsrA is H-NS^40^. We showed that deleting *dsrA* in the rpsD* strain restored motility (Figs 5 and 8A). In the absence of *hns*, deleting *dsrA* no longer increased motility of rpsD* cells (Fig. 8A). In contrast, deleting *rpoS* did not completely prevent the rescuing effect of *dsrA* deletion (Fig. 8A). To dissect the roles of the RpoS and H-NS pathways in regulation of motility by DsrA, we further took advantage of previously reported DsrA mutants that specifically impair regulation of *rpoS* (*dsrA* *R) or *hns* (*dsrA* *H)^41^. In the complementation assay, overexpressing WT DsrA or DsrA *R, both of which are able to inhibit H-NS activity, substantially reduced motility of the WT Δ*dsrA* strain (Fig. 8B). In contrast, overexpressing DsrA *H, which does not directly affect the H-NS pathway, showed only a minor decrease in motility (Fig. 8B).

DsrA regulates H-NS at the translational level^42^. In line with this, we found that the mRNA level of *hns* was unchanged by the rpsD* mutation (Fig. S2A). However, the activity of an H-NS repressed promoter (*hdeA*) increased significantly in the rpsD* strain (Fig. S2B), suggesting that the overall H-NS activity is lowered by the rpsD* mutation. In addition, the mRNA levels of *flhDC* were downregulated in the rpsD* strain (Fig. 4A), which is consistent with previous reports that H-NS stimulates transcription of *flhDC*^43^. Our results therefore suggest that DsrA regulates flagellar synthesis and motility mainly through the H-NS pathway.

## Discussion

Bacteria utilize flagella for movement in the environment. Flagella are also used as bacterial mechanosensors to initiate biofilm formation^44^, and are important for virulence in many bacterial pathogens^32^. On the other hand, biosynthesis and functioning of flagella consume substantial cellular resources^45^, and flagella also activate the host immune response that inhibits and kills invading bacteria^46^,^47^,^48^. Flexible modulation of flagellar synthesis is thus important for bacterial adaptation to frequently changing natural environments. In this study, we demonstrate that reducing translational fidelity leads to reduced flagellar synthesis and loss of motility in *E. coli*. We have previously shown that reduced translational fidelity activates the general stress response, promoting bacterial survival under stress conditions^29^. Suppressing flagellar synthesis would allow cellular resources to be conserved for essential activities to maintain cell viability, e.g., synthesis of stress response effector proteins.

We show that mistranslation suppresses flagellar synthesis and motility through enhanced transcription of DsrA. DsrA is a small RNA found in multiple Gram negative bacteria, including *Escherichia*, *Salmonella* and *Shigella*. DsrA RNA level is significantly increased at low temperatures due to both increased transcription and decreased degradation^39^, and temperature regulation of *dsrA* transcription depends on complex promoter architecture^49^. Our results show that transcription driven by *dsrA* promoter is enhanced in the error-prone strain (Fig. 7). To date, the only known transcriptional regulator of DsrA is LeuO, which represses DsrA transcription^50^. In our previous RNA sequence results^29^, LeuO mRNA level is increased in the rpsD* strain compared to the WT. Exactly how DsrA transcription is regulated by mistranslation remains to be clarified in the future. It is likely that another unknown transcriptional regulator of DsrA is affected by global protein mistranslation, e.g., through stabilization of a transcriptional activator due to titration of available proteases by an increased level of mistranslated proteins. It is also possible that mistranslation causes LeuO to misfold and lose its activity.

Small RNAs have been shown to regulate motility via diverse mechanisms^51^. Our data suggest that the effect of DsrA on bacterial motility requires H-NS instead of RpoS. DsrA blocks synthesis of H-NS protein by base pairing with the translational start site of its mRNA^42^. Increased expression of DsrA in the error-prone strain is thus expected to lower H-NS activity. We have used an H-NS repressed promoter *hdeA* as a reporter to test the activity of H-NS and show that the H-NS activity is suppressed in the rpsD* strain compared to the WT (Fig. S2). H-NS regulates a large number of genes, including activation of *flhDC* transcription^40^,^43^. A recent study also suggests that H-NS influences bacterial motility via FlhDC-independent pathways^52^. We show that overexpression of FlhC is sufficient to restore motility of the error-prone strain (Fig. S3), suggesting that mistranslation suppresses motility mainly through downregulation of *flhDC* in a process that requires DsrA and H-NS. Collectively, our results have revealed a previously unknown linkage between translational fidelity and flagellar synthesis, which may play an important role in bacterial adaptation to ever changing environmental conditions.

## Materials and Methods

### Strains, plasmids, growth conditions and reagents

Strains and plasmids used in this study are listed in Table S1, and the oligos are listed in Table S2. *E. coli* was grown in Lennox broth (LB) at 37 °C with agitation unless otherwise indicated. Antibiotics were used at the following concentrations: ampicillin (Amp), 100 μg/ml; chloramphenicol (Chl), 25 μg/ml. Antibiotics and other chemicals were purchased from Sigma-Aldrich (St. Louis, MO), and RNase-free DNase I was from Thermo Scientific (Rockford, IL).

### Genome engineering of bacterial strains

All strains used in this study are derivatives of *E. coli* K-12 strain MG1655 (WT), which was obtained from The *E. coli* Genetic Stock Center at Yale University. All in-frame gene deletion mutants were constructed as described using chloramphenicol as the resistance marker^53^. All mutants were verified by PCR, and the antibiotic resistance marker was subsequently removed from the deletion strains using plasmid pCP20, which was cured at 42 °C afterwards. The marker-free deletion mutants were verified by both loss of resistance and PCR.

The fusion of 3 × FLAG tag at the 3′ end of *flhD* was conducted as follows. A cassette containing the toxin encoding gene *ccdB* under control of araBAD promoter and a kanamycin resistance gene (Ranquet *et al*., submitted, deposit patent number: FR11/60169, 08/11/2011, UJF/BGene) was amplified from template genomic DNA of CR201 strain (obtained from N. De Lay) using the primers FlhD-KN1 and FlhD-CCDB1, and introduced into chromosome by λ red recombinase-mediated gene replacement. The *kan*-*ccdB* cassette fused with *flhD* in the chromosome was then replaced with the gBlock fragment of 3 × FLAG tag (FlhD-FLAG, synthesized from Integrated DNA Technology). The successful recombinants (YF56 and YF57) were obtained by selection for growth in the presence of arabinose (1%) and verified by PCR.

### Electron microscopy

Overnight culture of bacteria were diluted 1:100 into fresh LB and grown to OD_600_ ~ 0.8 at 25 °C with agitation. Cells were collected and washed in 0.1 M NaCl, and resuspended in phosphate-buffered saline. To examine cells by electron microscopy, 7 μl of culture was placed onto carbon-coated nickel grids (Electron Microscopy Sciences) for 1 minute, washed three times with sterile water and then negatively stained with 0.2% uranyl acetate for 30 seconds. The samples were visualized using a JEOL JEM-1400 electron microscope. Cells were randomly selected to count the number of flagella.

### Swimming motility assay

Overnight culture of bacteria were diluted 1:100 into fresh LB and grown to OD_600_ ~ 0.8 at 25 °C with agitation. All cultures were normalized to the same OD_600_ before being spotted on freshly made tryptone broth (10 g/L of tryptone and 5 g/L of NaCl) plates containing 0.25% agar. For strains harboring plasmids, appropriate antibiotics were added into the tryptone broth motility plates. Plates were incubated at 25 °C overnight before taking pictures and measuring diameters of spots. The quantitative results represent the percentage of the diameter compared to that of the WT strain on the same plate.

### Quantitative reverse transcription-PCR

Mid-log phase cells grown in LB medium at 25 °C was normalized to the same OD_600_ and harvested. Total RNA was extracted using hot phenol and residual chromosomal DNA was removed as previously described^54^, except that glycogen was used to precipitate RNA samples. To test RNA degradation, freshly made rifampicin (250 μg/ml final concentration) was added into normalized bacterial cultures to fully stop transcription at time zero.

Reverse transcription and quantitative PCR were performed using the iScript cDNA Synthesis Kit and the SsoAdvanced Universal SYBR Green Supermix Kit (Bio-Rad, Hercules, CA, USA) according to the manufacturer’s instructions. 16S rRNA was used as an internal reference for normalization. The ΔΔ*C*_*t*_ method was used to obtain the fold change of target genes in the mutant strains compared to those in the WT strains.

### Determination of FlhD protein expression

To determine expression of the FlhD, a 3 × FLAG tag was fused at the C terminal of *flhD* right before the stop codon in both WT and rpsD* strains. Mid-log phase cells grown in LB medium at 25 °C was normalized to the same OD_600_ and harvested. Same volume of bacterial cultures was used to prepare total protein using the standard trichloroacetic acid/acetone protein precipitation protocol. For sample preparation to test FlhD degradation, freshly made chloramphenicol (100 μg/ml final concentration) was added into normalized bacterial cultures to fully stop translation at time zero, and same volume of cultures was collected for protein preparation at specific time point. Western blot was performed according to standard procedures^55^ using a primary anti-FLAG antibody.

### Bacterial fluorescence protein and lacZ reporter

To measure the fluorescence intensity of reporter strains, overnight culture of bacteria was diluted to 0.01 OD_600_ in LB. Cells were further grown in 96-well plates incubated at 25 °C in the plate reader (BioTek) with shaking. Both OD_600_ and fluorescence were measured at 15 minute intervals for a total of 20 hours. Strains carrying pZS*11 were used as positive controls to eliminate the differences of protein synthesis rate between different strains. For *lacZ* reporter measurement, β-galactosidase assay was conducted as described^24^.

## Additional Information

**How to cite this article**: Fan, Y. *et al*. Reduced Protein Synthesis Fidelity Inhibits Flagellar Biosynthesis and Motility. *Sci. Rep.* **6**, 30960; doi: 10.1038/srep30960 (2016).

## Supplementary Material

Supplementary Information
