# Supplementary material for: Reduced Protein Synthesis Fidelity Inhibits Flagellar Biosynthesis and Motility
Source: Sci Rep. 2016 Jul 29;6:30960. doi: 10.1038/srep30960 (PMC4965754; doi:10.1038/srep30960)
Supplement: Supplementary Information [file srep30960-s1.pdf]

Reduced Protein Synthesis Fidelity Inhibits Flagellar Biosynthesis and Motility

Yongqiang Fan[1], Christopher R. Evans[1], and Jiqiang Ling[1, 2]*

Table S1. Strains and plasmids used in this study.

Strain or plasmid	Description	Origin
**Strains**		
WT	*E.coli* K-12 MG1655 (F-, λ$^-$, *rph-1*)	Lab Stock
*rpsD** (YF12)	*rpsD* I199N in chromosome	[1]
*rpsD** Rev (YF29)	*rpsD** reverted to WT	[1]
rpsD/L** (YF5)	*rpsD* I199N; *rpsL* K42N	This study
YF56	WT *flhD::3×FLAG*	This study
YF57	*rpsD* flhD::3×FLAG*	This study
YFK1G6	*rpsD* ΔrpoS*	This study
YFK2F4	*rpsD* ΔdsrA*	This study
YFK2F1	WT *ΔdsrA*	This study
YFK3C5	*rpsD* ΔdsrA/Δhns*	This study
YFK3C6	*rpsD* ΔdsrA/ΔrpoS*	This study
YFK3B6	*rpsD* Δhns*	This study
YFK3B5	WT *Δhns*	This study
CR201	*cya::kan ccdB*	C. Ranquet
**Plasmids**		
pKD46	Amp^r; repA101ts; *exo*, *bet*, *gam*; tL3; p*araB*	[2]
pKD13	Chl^r; *FRT-cat-FRT*	[2]
pCP20	Amp^r, Cm^r; cI857 P_R*::flp* pSC101 *oriTS*	[2]
pBR-plac	Amp^r; *lac* promoter-based expression vector	[3]
pDsrA	Amp^r; expresses WT *dsrA* from a *lac* promoter	[4]
pDsrA*H	Amp^r; expresses *dsrA*H* from a *lac* promoter	[5]
pDsrA*R	Amp^r; expresses *dsrA*R* from a *lac* promoter	[5]
pZS*11	Amp^r; SC101* *ori*; expresses *yfp* from *LtetO-1* promoter	[6]
pZS*-pflgB	Amp^r; SC101* *ori*; expresses *lacZ* from *flgB* promoter	This study
pZS*11-pdsrA	Amp^r; SC101* *ori*; expresses *yfp* from *dsrA* promoter	This study
pMSs201-phdeA	Kan^r; expresses *gfp* from *hdeA* promoter	[7]

Table S2. Oligos used in this study.

Oligos	Sequence (5' to 3')
rpsD I199N	T*G*T*G*TCCTCTCTTTGGTACTAAGCTTTACTTGGAGTAAAGCTCGACGTTAAGGT GTTCGTTAATGTCCGCAGACAGATCAGAACGCTCCG
rpsL K42N	T*C*A*G*ACGAACACGGCATACTTTACGCAGCGCGGAGTTCGGTTTGTTTGGAGTG GTAGTATATACACGAGTACATACGCCACGTTTTTGCG
flgK-F1	AGCTTCACGCTGAAACCAGT
flgK-R1	CCCACCGTTTTACTGTTGCT
fliA-F1	CGAACGCTATGACGCCCTAC
fliA-R1	TGCCCTATTGCCTGTGCC
flgB-F1	GTGGTTGCACTGACGATGAC
flgB-R1	CAGGCTGTTATCGGCAAACT
fliF-F1	TCCTGTCGCCTATTGTTGG
fliF-R1	CGATACTGTTCTTCCGTTTGT
16s-F4	CACAAGCGGTGGAGCAT
16s-R4	CTGGCAACAAAGGATAAGG
flhD-F1	CTTGCACAGCGTTTGATTGT
flhD-R1	GATGCCGGTATGAATTTGCT
flhC-F1	CCCACAAGCAGAAGAAGG
flhC-R1	ATGGCGGTTGACATAAGC
FlhD-KN1	CCAGCAAATTCATACCGGCATCATGCTCTCAACACGCTTGCTGAATGATGATAGGA ACTTCAAGATCC
FlhD-CCDB1	CACTCATGATCAGGCCCTTTTCTTGCGCAGCGCTTCTTCAGGCTGATTAATTATATT CCCCAGAACATCAGG
FlhD-FLAG (gBlock)	CCAGCAAATTCATACCGGCATCATGCTCTCAACACGCTTGCTGAATGATGGACTAC AAAGACCATGACGGTGATTATAAAGATCATGATATCGACTACAAAGATGACGACGA TAAATTAATCAGCCTGAAGAAGCGCTGCGCAAGAAAAGGGCCTGATCATGAGTG
dsrA-P1	TGGCGAATATTTTCTTGTCAGCGAAAAAAATTGCGGATAAGGTGATGAACATGGGA ATTAGCCATGGTCC
dsrA-P2	TCATGACTTCAGCGTCTCTGAAGTGAATCGTTGAATGCACAATAAAAAAATGTAGG CTGGAGCTGCTTCG
rpoS-P1	CTTTTGCTTGAATGTTCCGTCAAGGGATCACGGGTAGGAGCCACCTTATGATGGG AATTAGCCATGGTCC
rpoS-P2	TGAGACTGGCCTTTCTGACAGATGCTTACTTACTCGCGGAACAGCGCTTCTGTAG GCTGGAGCTGCTTCG
pZS*-flgB-lacZ_F	TCGTCTTCACCTCGAGTTAACGGACTGAACAGTATC
pZS*-flgB-lacZ_R	GTAATCATGGTCATGGTACCTCCGCAGGTATCAAAATTCT
dsrA-F1	CATCAGATTTCCTGGTGTAACG
dsrA-R1	CTGAGGGGGTCGGGATGAAAC
PdsrA-pZS-infu-IF	TCGTCTTCACCTCGAGAAAACATAGTCGCGCAGTAC
PdsrA-pZS-infu-IR	CAAAATTATTGAATTCTCATCACCTTATCCGCAATTTTTTTCG
hns-P1	ATTATTACCTCAACAAACCACCCCAATATAAGTTTGAGATTACTACAATGATGGGAA TTAGCCATGGTCC
hns -P2	GATTTTAAGCAAGTGCAATCTACAAAAGATTATTGCTTGATCAGGAAATCTGTAGG CTGGAGCTGCTTCG
hns-F1	TGAAGAGCGCACTCGTAAAC
hns-R1	TAACGGCAGCAAGGCTATTC

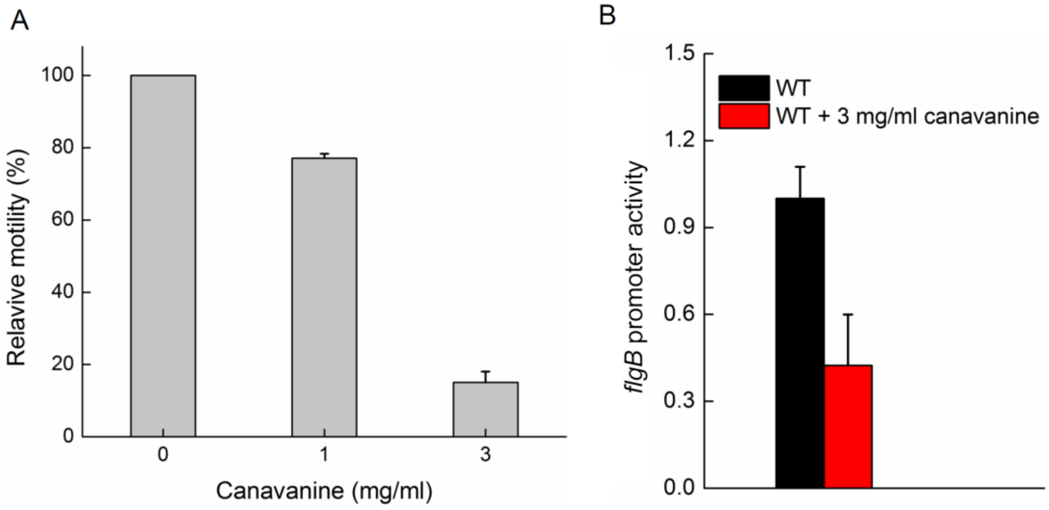

Fig S1. Canavanine decrease motility (A) and *flgB* promoter activity (B) in wild-type *E. coli* MG1655. The promoter activity of *flgB* is normalized with the activity of a constitutive *tet* promoter under the same growth condition.

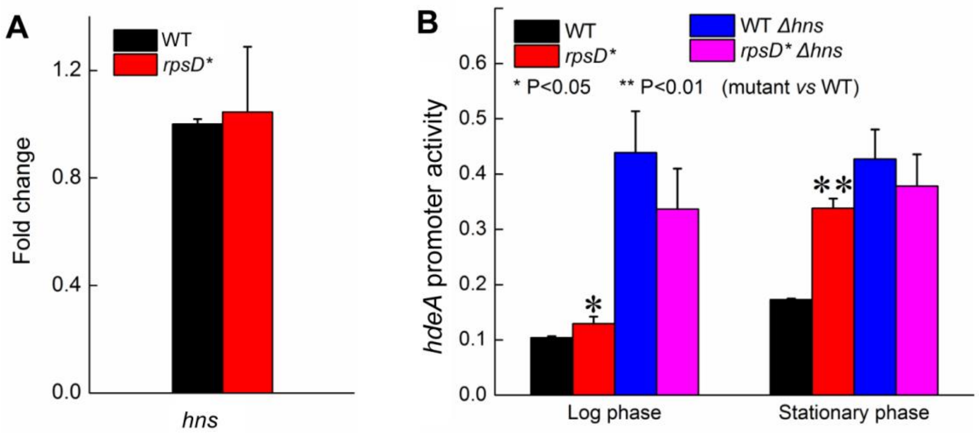

Fig S2. Expression and activity of H-NS. (A) The *rpsD** mutation did not affect the mRNA level of H-NS. (B) H-NS represses transcription of *hdeA* promoter. The promoter activity of *hdeA* is increased by deleting *hns* or the *rpsD** mutation, suggesting that the *rpsD** mutation reduces the activity of H-NS. The results are the average of at least three repeats with error bars indicating standard deviations.

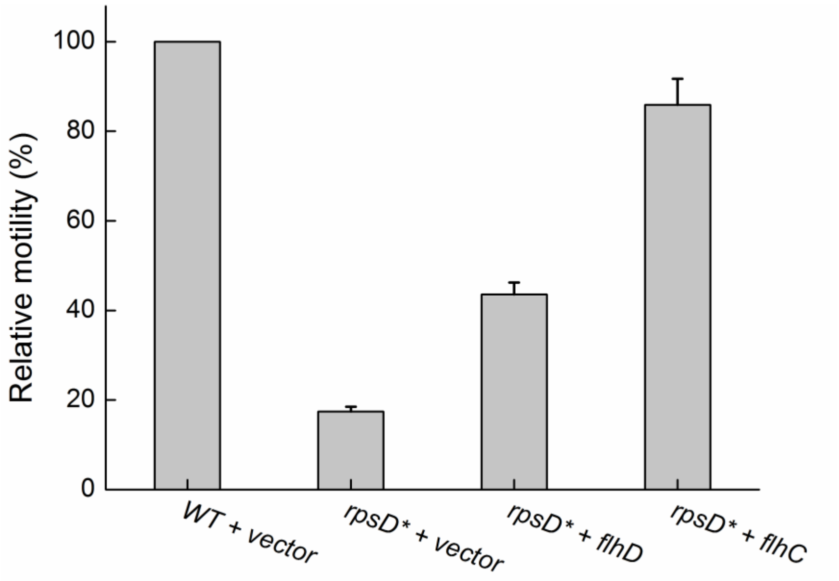

Fig S3. Complementation of motility defect caused by *rpsD mutation.** Overexpressing *flhD* or *flhC* rescues motility of the *rpsD** cells.

References

1. Fan Y, Wu J, Ung MH, De Lay N, Cheng C, Ling J. Protein mistranslation protects bacteria against oxidative stress. *Nucleic acids research* **43**, 1740-1748 (2015).

2. Datsenko KA, Wanner BL. One-step inactivation of chromosomal genes in Escherichia coli K-12 using PCR products. *Proceedings of the National Academy of Sciences of the United States of America* **97**, 6640-6645 (2000).

3. Guillier M, Gottesman S. Remodelling of the Escherichia coli outer membrane by two small regulatory RNAs. *Mol Microbiol* **59**, 231-247 (2006).

4. Sledjeski D, Gottesman S. A small RNA acts as an antisilencer of the H-NS-silenced rcsA gene of Escherichia coli. *Proceedings of the National Academy of Sciences* **92**, 2003-2007 (1995).

5. Lease RA, Cusick ME, Belfort M. Riboregulation in Escherichia coli: DsrA RNA acts by RNA: RNA interactions at multiple loci. *Proceedings of the National Academy of Sciences* **95**, 12456-12461 (1998).

6. Subramaniam AR, Pan T, Cluzel P. Environmental perturbations lift the degeneracy of the genetic code to regulate protein levels in bacteria. *Proceedings of the National Academy of Sciences of the United States of America* **110**, 2419-2424 (2013).

7. Silander OK*, et al.* A genome-wide analysis of promoter-mediated phenotypic noise in Escherichia coli. *PLoS Genet* **8**, e1002443 (2012).
